# How to Keep Well

**Published:** 1890-10-18

**Authors:** 


### The Doctor's Pulpit.

### HOW TO KEEP WELL.

After the Psalms, there is probably no more interesting reading to those who have attained middle age than the book of Ecclesiastes. Solomon truly says there is nothing better for a man than that he should eat and drink, and that he should make his soul enjoy good in his labour. There was never a time in the history of the world when there were more prophets and teachers of what to eat, drink, and avoid than in the present day. The effect of the alteration which has taken place in public opinion concerning eating and drinking is very forcibly illustrated by a comparison of those who formerly filled the carriages in the Lord Mayor's Procession with those who occupy them to-day. Last year we heard a remark by a disappointed member of the public in the crowd which forcibly illustrated this. He said to a companion, speaking of the occupants of the carriages, "Well, they might be total abstainers if they were judged by their lean looks. I calls 'em the exact opposite of what an alderman ought to be." No doubt early dinners and indulgence in heavy potations after them exercised a marked effect on the outward appearance of the man. Nowadays, even where the members of a City Company belong to the old school, the amount of food, and especially of wine, consumed is very small compared with what it was forty or even twenty years ago. We rejoice that this should be the case, and we have no doubt that this improvement in the habits not only of aldermen, but of ordinary men, has tended to develop the tendency of giving more and more largely each year from corporate funds to charitable and public purposes.

Everybody, even if he wishes to be so—and this is by no means the general feeling—cannot, however, be an alderman, though all must eat and drink. We have, therefore, as individuals, to bear continuously in mind that life is not to live, but to be well. Every person, it has been well said, who has attained forty years of age, is either a fool or his own best physician. That is to say, not that men or women should dose themselves with physic when seriously ill, or at any time for that matter, if they are wise (medical treatment being a matter too serious to trifle with, and one which should never be adopted unless under a doctor's directions); but that they should take the trouble to observe and test their habit and constitution, and so learn what food, what amount of exercise, and how much sleep is best for them. They also know, or should know, perfectly well how much wine or alcohol in any form is good for them, or whether on the whole they can work better or be better in bodily health by avoiding alcohol altogether. In the same way a man learns from experience whether tobacco is helpful or the reverse, and many a busy worker knows that under certain conditions an amount of tobacco may be consumed with advantage, which at other times would produce congestion of the throat or other serious discomfort. We would, therefore, urge the young to consider these things in the days of their youth, and to realise the advantage of always feeling well. If they do this, then those who are self-respecting and who wish to be useful members of society will avoid even a limited indulgence in tobacco or alcohol if they find they are better without one or the other.

Some people, and especially some women, deliberately undermine their constitution, and gradually grow into confirmed invalids. Not only is this wrong and foolish, but it is unwholesome and wicked. Many a woman has some ailment which troubles her, and which she will not speak about to her dearest friend, much less to a professional man. We have known cases where the want of a little attention to diet, and the lack of the exercise of sufficient courage and energy to consult a medical man when the trouble first became oppressive, have led to most serious consequences. It is not right for a wife to be continually fretful, ill at ease, and anything but a companion to her husband, because she allows herself to suffer say, from obstinate constipation or some kindred ailment, the consequences of which are not only hurtful but unpleasant. Why women, and especially young women, are so foolish it is difficult to say. They seem to think that it is indelicate to mention their ailments to a doctor, or even to their husband, or they have been so accustomed to have their own way and to be sheltered by their parents from unpleasantness and trouble of all kinds that they lack the energy, when thrown on their own resources, to act for themselves should occasion arise. The first excuse is now inadmissible because there are at the present time a number of excellent and capable women who are also qualified medical practitioners. But apart from this we wish distinctly to urge that it is morally wrong for any woman to conceal her ailments to the detriment of her own health and to the discomfort of those with whom she has to live. We have not the smallest doubt that it is an offence against the Creator to adopt so wrong a course, and it is difficult to understand how any woman can allow herself to commit a sin of this kind for years without realising what she is doing.

Take, again, the question of exercise which is often a very pressing one to a business man. Exercise is essential to health, and health is greatly essential to happiness. Anyone who has much head work to do, must take a sufficient amount of exercise daily. No doubt, in these days of competition, so long as a man can manage to do his work without being laid up, he will very often grudge the hour or half-hour which exercise demands at his hands. Some people, and even the preacher himself, may realise how essential exercise is, but from circumstances they may take far too little. The consequences of non-exercise to a town resident are increased girth, a tendency to irritability, headache, and other symptoms which are sure to end sooner or later in sleeplessness or breakdown. Besides experience shows that a man or woman who works hard and remains indoors during the greater part of each day without regular exercise, is certainly preparing the way to the development of some chronic ailment which may make life miserable, and which will almost certainly shorten life, and so limit individual usefulness.

We hope our readers will really consider this question very seriously, and that they will provide that every day a certain defined time shall be always given to exercise. Walking, if an hour can be spared is as good a form of exercise as can be taken. Those who can afford it probably keep a horse on Lord Palmerston's principle that the outside of a horse is the best thing for the inside of a man. In most town houses there is a sufficient space at the back to afford ample room for a badminton court, which can be permanently marked out in concrete on the gravel at a small expense. In the winter months, during some portion of each day, the weather will usually permit of a game lasting half-an-hour, which will give an amount of exercise few can realise who have not tried it. Whatever the form of exercise may be, however, daily exercise must be taken, or individual usefulness and health must suffer. It is uneconomical and selfish for a hard worker to allow his business to encroach upon the time which ought to be set apart absolutely for physical exercise.

